# Biomechanical Multipurpose Miniscrew Strategy for Simultaneous Distalization in Class II Patients—The BiGa System

**DOI:** 10.3390/biomimetics9050305

**Published:** 2024-05-20

**Authors:** Gabriele Di Carlo, Guglielmo Biondi, Ivan Gazzola, Matteo Saccucci

**Affiliations:** 1 Department of Oral and Maxillo-Facial Sciences, Sapienza University of Rome, Viale Regina Elena 287a, 00161 Rome, Italy; gabriele.dicarlo@uniroma1.it; 2 Private Practice, 47121 Forlì, Italy; drguglielmobiondi@gmail.com; 3 Private Practice, 31033 Castelfranco Veneto, Italy; gazzivan@gmail.com

**Keywords:** anchorage procedure, orthodontic, malocclusion, angle class II, miniscrew

## Abstract

An efficient treatment plan using a temporary anchorage device should be built following the principle of reducing the number of tads to obtain a multiple biomechanical advantage. The following case report concerns the Biga system, a strategy that supports orthodontists during class II corrections and vertical control through treatment. A 12-year-old girl with a high angle of skeletal class II was selected. A novel biomechanical strategy was effectively applied using two tads on the upper arch to obtain sequential distalization of the upper teeth and to correct the lower arch spee curve using third-class elastics. Eventually, on the same tads, a double cantilever was applied to control the overbite and intrusion during incisors’ retraction. The Biga system is an easy biomechanical strategy that ensures the three-dimensional control of treatment mechanics in class II patients.

## 1. Introduction

Molar distalization can be achieved through the use of extra- and intra-alveolar forces. The disadvantage of the use of extra-alveolar forces is the need for some degree of cooperation from the patient, made complex by the fact that the use of extra-alveolar appliances results in unacceptable esthetics. For this reason, the use of intraoral forces has become established, which, however, has the disadvantage of offloading orthodontic forces onto tooth elements such as the incisors and premolars, where such forces cause undesirable effects. When trying to prevent undesirable effects, the choice of skeletal anchoring is essential. In particular, for the correction of class II malocclusions, miniscrews play an essential role because their use reduces the need for extractions in cases of camouflage of the second class. At the same time, laboratory procedures are simplified, such as in cases where the use of a distal jet and pendulum is recommended. This is especially true when using miniscrews in the vestibular area. Unfortunately, the use of temporary anchorage devices (tads) is not enough per se to gain complete control over side effects. In fact, miniscrews are the best system to produce maximum anchorage. However, on their own, without adequate biomechanical control, they are not able to guarantee a well-defined and controlled retraction of the incisors. Both in sliding mechanics and in other techniques, it is essential to adjust the following factors to minimize sudden side effects such as the loss of torque control of the upper incisors, which can lead to the appearance of a deep bite or lateral open bite caused by tipping of the anterior and posterior teeth and increases in friction/binding forces, especially when sliding mechanics are applied [1]. Moreover, an efficient treatment plan using a temporary anchorage device should be built following the principle of reducing the number of tads to obtain a multiple biomechanical advantage. Nowadays, the strategy of distalizing teeth using palatal anchorage is a well-accepted approach to mechanics. The placement of miniscrews in distalization cases sees the orthodontic market leaning toward the palatal location. This may lead the orthodontist to defocus on the possibility of taking advantage of an opportunity to position miniscrews within the buccal segment. Undoubtedly, the palate has a good amount of bone and keratinized tissue. Another advantageous factor is the possibility of placing the anchorage system away from the roots of the dental elements. Nevertheless, the increased complexity of insertion procedures and the associated increase in costs to make both the insertion template and the device associated with the miniscrews themselves should not be underestimated. The use of miniscrews in the buccal site, although it has limitations due to the presence of the roots of the dental elements, is, in fact, characterized by greater convenience in their insertion and has biomechanical advantages in preventing mesial rotation of molars during the distalization procedure. The latter is a limitation of palatal distalizers, which is also associated with the frequent presence of molars already mesialized in the second class [2].

The aim of the following article is to describe and demonstrate a new system for non-extraction treatment of class II malocclusions that combines the use of tads and the principles of edgewise mechanics [3]. The following biomechanical system is conceptualized to guarantee upper molar distalization, control of the occlusal plane’s canting, as well as torque upper incisor control in space closure. At the same time, the system can control lower incisor flaring during the spee curve correction and recovery of the incisor mandibular plane angle when required.

## 2. Case Report

**Figure 1:**
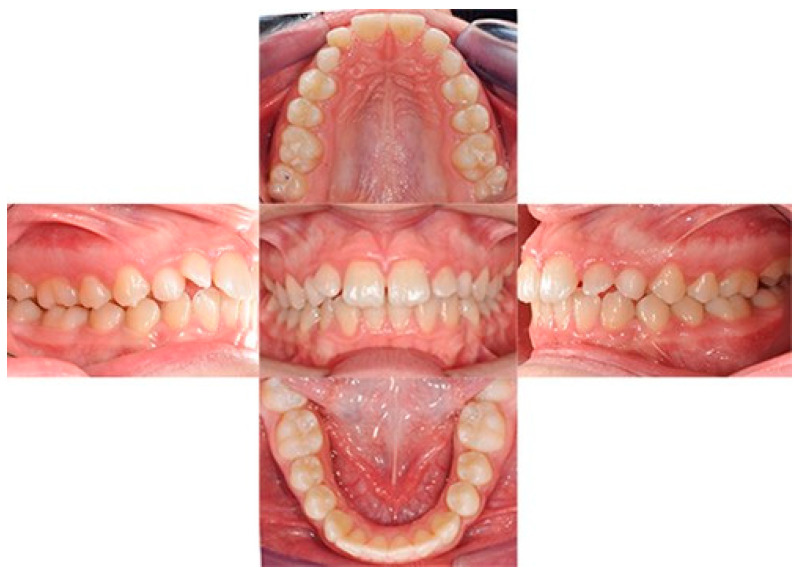
Initial intraoral photos.

**Figure 2:**
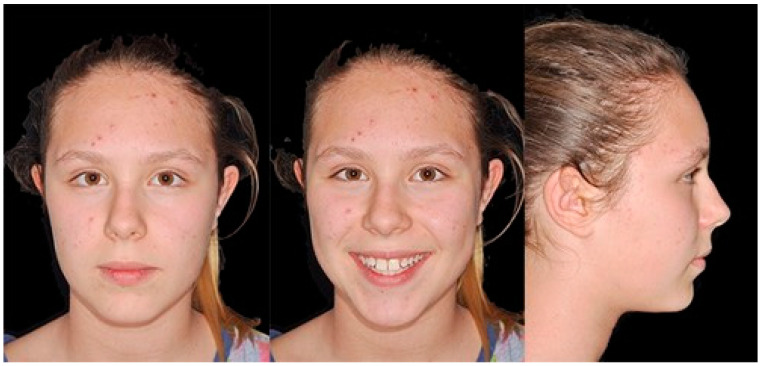
Initial extraoral photos.

**Figure 3:**
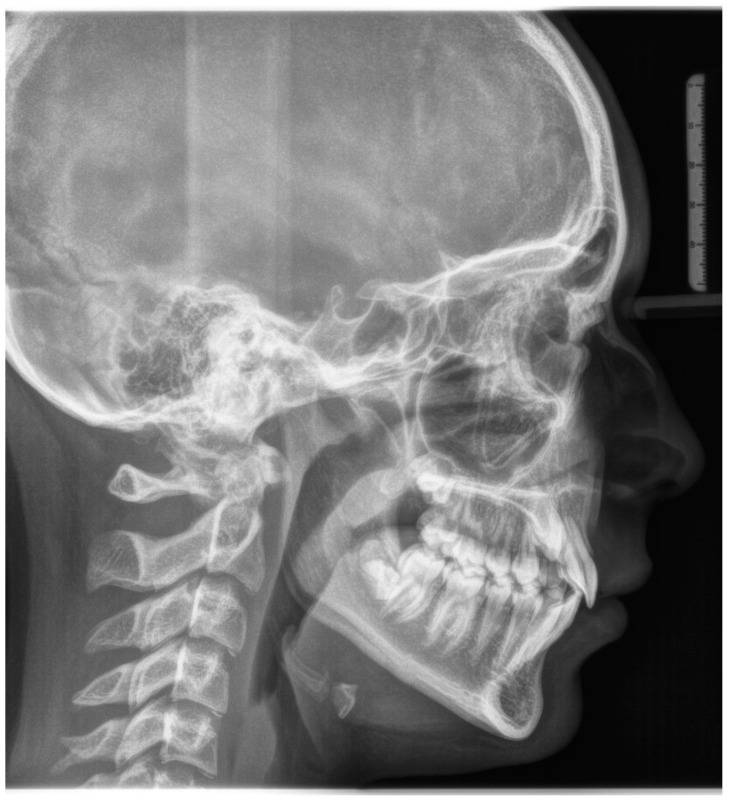
Initial lateral cephalogram.

A 12-year-old girl with the second skeletal class (ANB 5), a high angle (FMA 27), and a facial index of 0.67 was selected. Soft tissue analysis showed a convex profile with a Z angle equal to 57 and an FMIA angle close to 55. The IMPA angle was 98. The analysis of the dental cast revealed a spee curve of 3.5 mm (Table 1). The occlusion presented a first molar class to the left, and a light second class to the right with 1 mm crowding in the lower jaw. The overjet was 0. The upper canines were impacted (Figure 1, Figure 2 and Figure 3). The patient’s chief complaint was altered aesthetic perception due to reduced dimensions of the deciduous canines. The treatment objectives were to retrieve space for upper canines’ eruption, level the spee curve, upright the lower incisors, and reduce the impact of the convex profile through occlusal vertical control to facilitate counterclockwise mandibular rotation.

### 2.1. Treatment Progress

The treatment sequence can be didactically divided into two parts: molar and premolar distalization, and canine and incisor distalization.

### 2.2. Molars’ and Premolars’ Distalization

The treatment sequence starts with sequential bonding of the upper arch with 0.17 × 0.22 SS. A passive distal tip is incorporated in the arch after the readout of the upper second molars.

After achieving the full bonding on the upper arch, two miniscrews (tads) are placed between the upper first molars and the second bicuspid, bilaterally. A 0.19 × 0.25 SS wire is shaped, adding a helical bulbous loop, bent lingually, flush to the second molar tube. At the same time, an active 7° lingual crown torque is placed on the second molar. A long spur with a mesial hook, just mesial to the bracket of the second bicuspid, is soldered. A metallic ligature is placed between the tads and the spurs.

**Figure 4:**
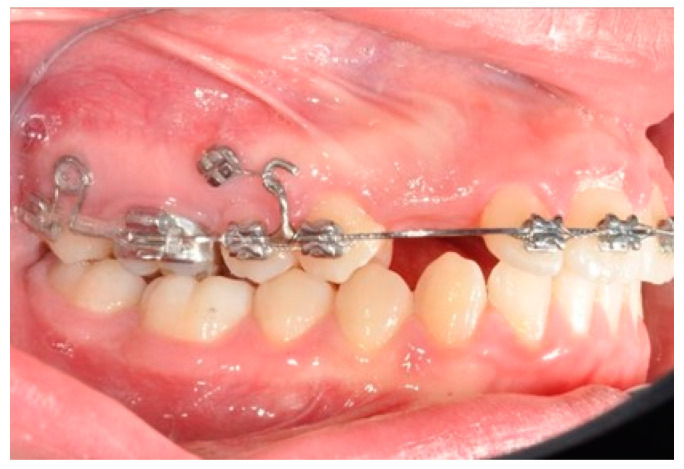
Molar and premolar distalization strategy.

**Figure 5:**
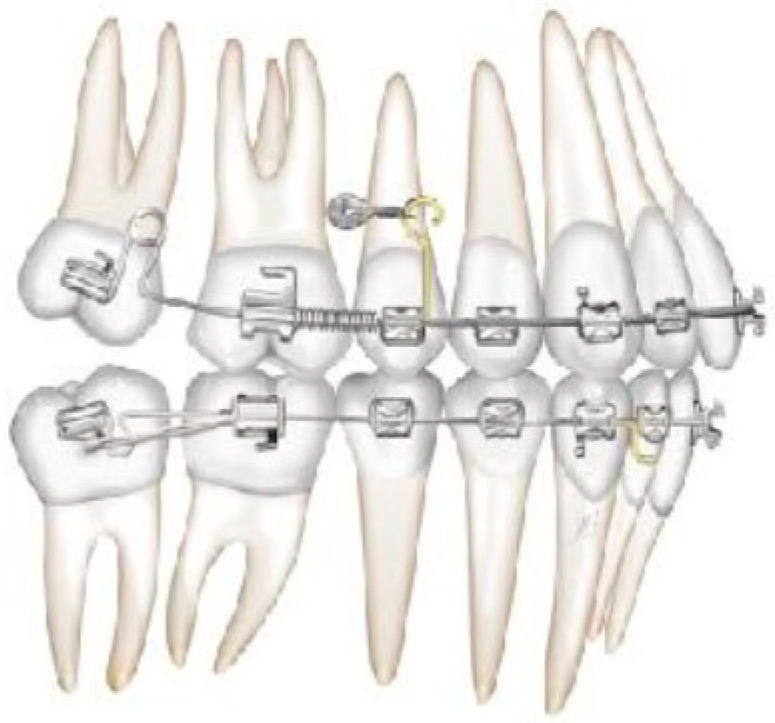
The first molars are distalized by using a simple open coil spring.

The helical bulbous loop produces space distally to the upper first molars. An open coil is added between the second bicuspid and the first molar in order to move to the distal second and first molar at the same time (Figure 4 and Figure 5). Eventually, an elastic module is placed between the hooks on the second molars and the first molars in order to finish the combined distalization of the upper first molars.

**Figure 6:**
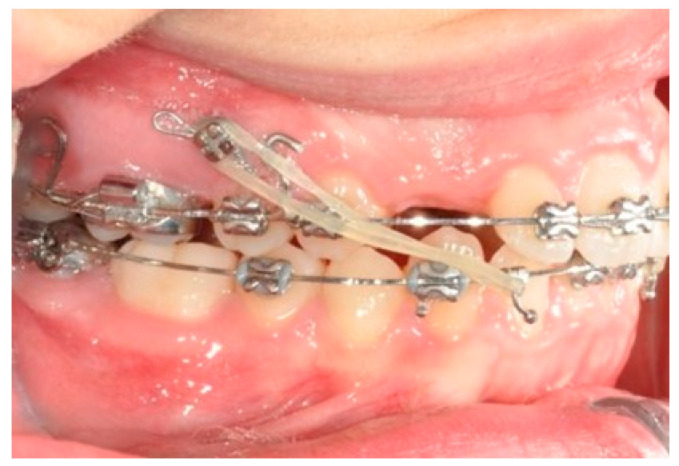
Molar and premolar distalization strategy: Hooks are placed between the laterals and the cuspids. Third-class elastics (1/4” 6 0z) are placed from the tads to the hooks.

After tad positioning, sequential bonding is applied on the lower arch. An 18 × 0.25 SS arch is shaped, adding a maximum 10° distal tip to the second molar. Hooks are placed between the laterals and cuspids. Third-class elastics (1/4” 6 0z) are placed from the tads to the hooks (Figure 6). The distal tip on the lower second molars will produce a gain of space between molars, with the space needed for the management of the spee curve and the recovery of IMPA.

Once the right uprighting and the tipback position of the second molar are achieved, the first molars are distalized by using an open coil spring (Figure 5).

**Figure 7:**
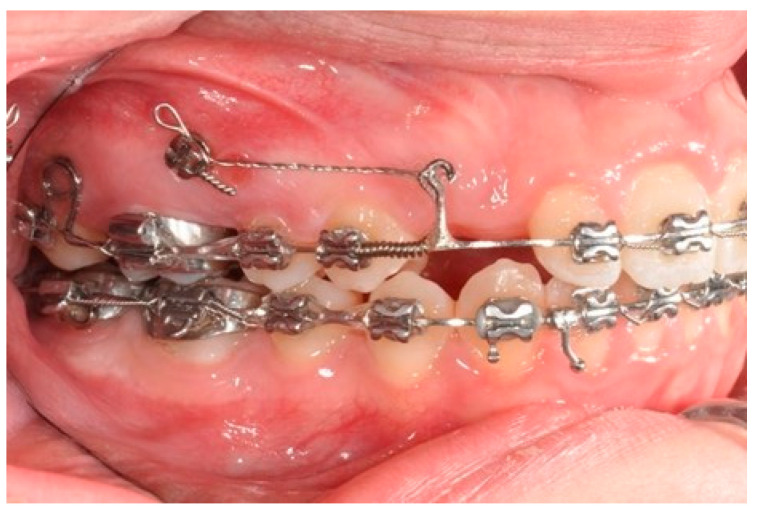
Molar and premolar distalization strategy: Power chain from the first molar (blocked with a metallic ligature with the second molar) to the second bicuspid and coil spring between the solder and the first bicuspid allow for the simultaneous distalization of the second and first bicuspids.

Once the upper first molars reach the hyper-first-class position, the tads are repositioned exactly mesial to the first molar. The solder distal to the bicuspid is removed and a new one is made distal to the ideal cuspid position. A power chain from the first molar (blocked with a metallic ligature with the second molar) to the second bicuspid and a coil spring between the solder and the first bicuspid allow for the simultaneous distalization of the second and first bicuspids (Figure 7).

**Figure 8:**
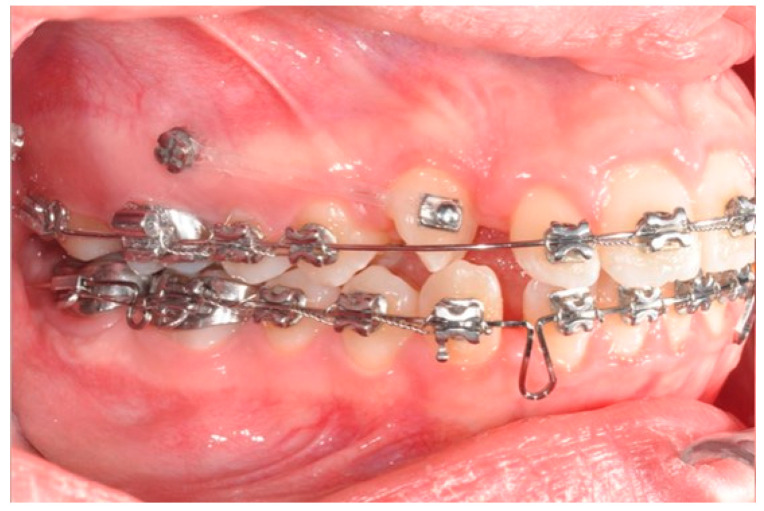
Power chain used to derotate and distalize the right upper canine.

On the lower arch, once the molars reach the hyper-first-class position, second and consequently first bicuspid distalization begin, produced by a power chain, and they proceed until reaching cuspid distalization (Figure 8).

This produces a space between the cuspid and the lateral, which will be fundamental to control and, when necessary, recovering the IMPA angle, through simple closing loops placed on 0.18 × 0.25 SS wire between the lateral and the cuspid, activated by tying the mesial leg of the omega loop to the second molar (Figure 8). In this case, on the upper arch, the distalization of the upper molars and the bicuspids produced a spontaneous eruption of the cuspid (Figure 8).

### 2.3. Canine and Incisor Distalization

**Figure 9:**
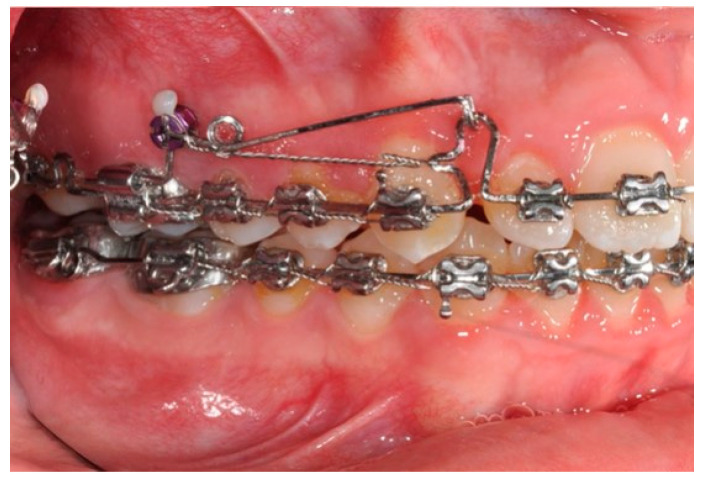
Canine and incisor distalization strategy: The cantilevers produce intrusion of the incisors and torque control during retraction.

**Figure 10:**
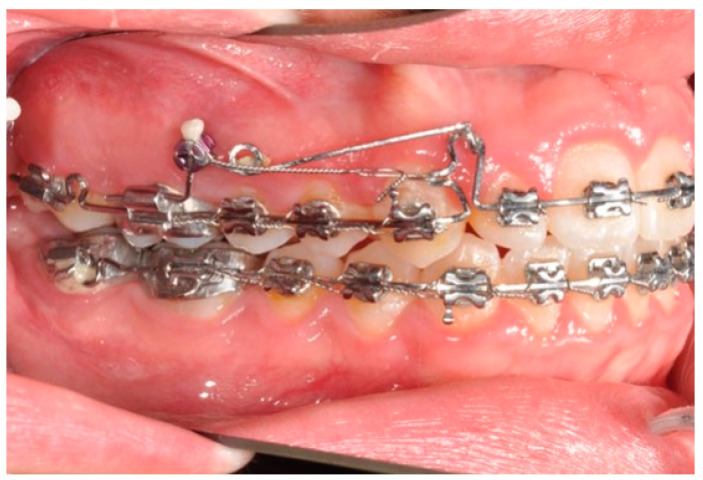
Canine and incisor distalization strategy.

In this case, right after the spontaneous eruption, a power chain from the right tad was used to derotate and distalize the right upper canine (Figure 8). On the upper arch, a 0.20 × 0.25 SS wire was prepared with modified “cactus-shaped” closing loops, with the aim to close the space between the canines and the laterals. The cactus loop has a double aim. First of all, it is used as a closing loop; activation is produced by tying the loop through a metallic ligature from the tads. Secondly, it serves as a hook for cantilevers. The cantilevers are made using 0.19 × 0.25 TMA. The retraction of the upper incisors is supported by two cantilevers applied distally on the tads. The cantilevers will support the mechanics in order to produce intrusion of the incisors and torque control during retraction [1] (Figure 9 and Figure 10).

**Figure 11:**
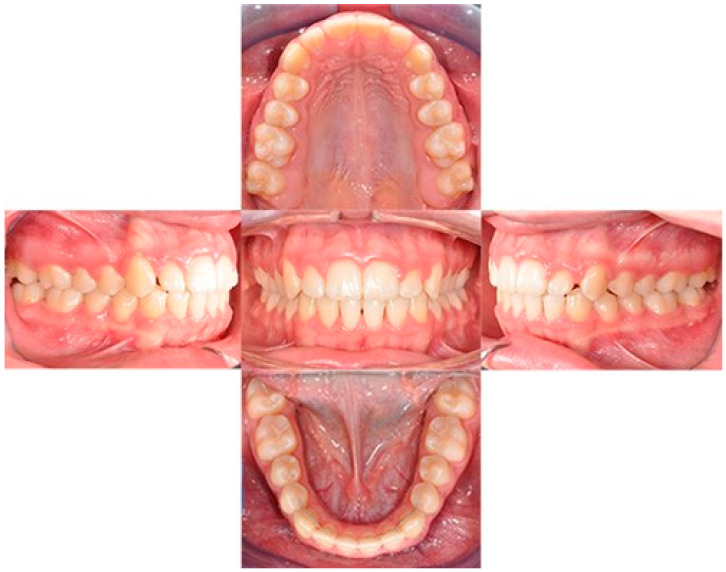
Final intraoral photos.

**Figure 12:**
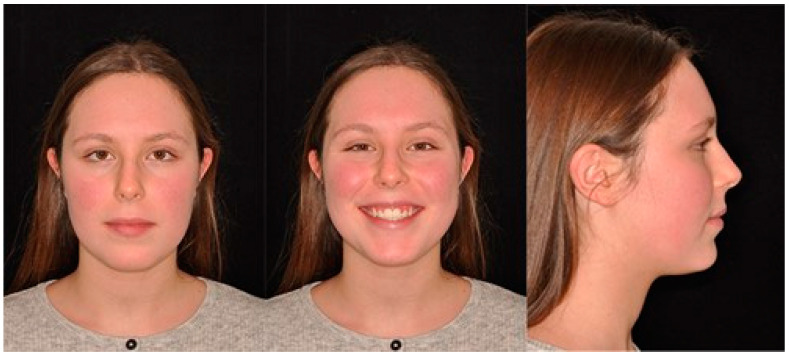
Final extraoral photos.

**Figure 13:**
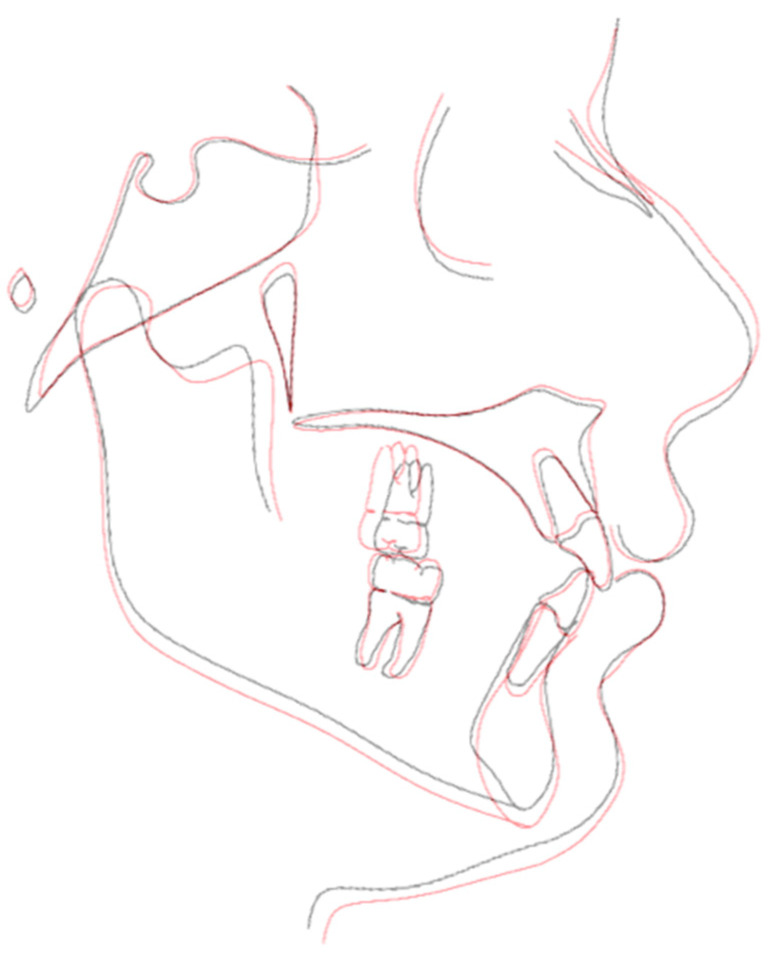
Superimposition.

The treatment duration was 26 months. The post-treatment facial photographs showed a nicely balanced and harmonious face. A class I occlusion was obtained, and a distal space resulted in the upper incisors due to Bolton discrepancy. Prosthetic restoration was programmed to complete the patient’s smile. According to Table 1, counterclockwise rotation of the mandible was achieved (Figure 11, Figure 12 and Figure 13).

## 3. Discussion

The Biga system is used to obtain a full soft tissue benefit or maintenance. In this case, the use of an uncontrolled Niti wire during alignment would have resulted in a loss of vertical control and post-mandibular rotation. This was not in accordance with the objectives of the treatment. In fact, although it would have been easy to achieve dental correction in the patient, the same could not be said of her soft tissues. In particular, the lower third of her face would have been damaged. In this regard, the maintenance of lower incisors’ positions and the vertical control of molars during distalization are essential to ensuring a patient’s facial balance [4,5].

The biomechanics of the distalization on the lower arch show us how by applying third-class elastics in the typical style direction of the Biga system, it is possible to obtain distalization of the molars and, at the same time, retroclination and extrusion of the incisors. According to the treatment plan, in this case, the maintenance of incisors’ positions was pivotal. In that respect, the addition of a tipback to the lower molars added an intrusive and vestibular force from the incisors, which controlled the anterior side effects, mainly developing the distalization of the molars [6].

The use of cantilevers on miniscrews is essential to maintain the class I molar relationship obtained after distalization. Indeed, a loss of molar anchorage not only affects the anterior positions of molars but also induces modifications to the overall vertical dimensions of the face [1]. While retracting the upper incisors using forces at the occlusal level, it is pivotal to control the moment produced by the force, which is responsible for the loss of incisor torque. The use of a cantilever produces a counteracting moment. This moment is also responsible for a certain amount of intrusion, which, in this case, was favorable. The distalization of molars can also be achieved through palatal anchorage [2]. Although this treatment strategy has proven to be efficient, the Biga system offers a valid alternative with a lower cost for the orthodontist and it represents a comprehensive treatment strategy for the dentition. Indeed, the main distalization techniques are presented as ends in themselves; however, in this article, an inclusive treatment strategy has been proposed that is not only aimed at producing distalization and controlling side effects. On the contrary, the Biga system is characterized by achieving several biomechanical advantages on both the upper and lower arches.

Limitations of this treatment strategy are the possibility of failure of the miniscrews and the potential need to reposition them during the course of therapy. The miniscrews should ideally remain stable during the application of a useful force, to allow displacement of the dental elements. The problem with stability lies primarily in the lack of osseointegration of the miniscrews into the alveolar bone compartment. Instead, the factor that ensures their stability is the ability to mechanically embed themselves in the bony compartment so that they can withstand mechanical loading. From this point of view, several factors must also be taken into account with respect to the selection of a patient suitable to be treated with miniscrews [7]. For example, the age of the patient must be considered since the risk of failure is higher in an adolescent patient than in an adult patient. Smoking has also been associated with a high risk of miniscrew loss. On the other hand, it is crucial in the management of these clinical cases to provide the patient with all the tools to be able to perform proper oral hygiene since this has been associated with a higher chance of keeping the miniscrews in place [8]. Another key factor is the site of insertion. In our case, miniscrews were inserted at the maxillary site, where adequate retentive capacity has been demonstrated [9], and in keratinized gingiva, the latter increasing the chances of success [7]. As demonstrated by several studies, appropriate consideration of these factors underpins a reliance on complex, multipurpose mechanics using miniscrews as anchors [10].

## Figures and Tables

**Table 1:** Cephalometrics value pre-post- treatment.

Cephalometrics Value Pre-Post-Treatment			
	Normal Value	Pre Tx	Post Tx
FMIA	67	55	57
FMA	25	27	26
IMPA	88	98	97
1-MxP	110	113	110
SNA	82	84	84
SNB	80	79	81
ANB	2	5	3
AO-BO	2 mm	2 mm	1 mm
OP	10	8	5
Z	75	57	75
PFH	45 mm	41 mm	44 mm
AFH	65 mm	62 mm	66 mm
INDEX	0.69	0.67	0.68

## Data Availability

The data for the original contributions presented in this study are included in the article, and further inquiries can be directed to the corresponding author.
